# Sex and ethnic differences in the waist circumference of 5-year-old children: Findings from the Millennium Cohort Study

**DOI:** 10.3109/17477166.2010.526224

**Published:** 2011-06

**Authors:** L J Griffiths, C Dezateux, T J Cole

**Affiliations:** MRC Centre of Epidemiology for Child Health, UCL Institute of Child HealthLondon, UK

**Keywords:** Children, ethnicity, waist circumference, central fatness

## Abstract

We examined sex and ethnic differences in central fatness, as assessed by waist circumference measurements, in 13 590 Millennium Cohort Study 5-year-olds. Measurements were expressed as z-scores based on reference data from the British Standards Institute. The cohort, especially girls, had larger waist circumference measurements than the reference population. Black children had larger waists, and children from other minority ethnic groups had smaller waists than White children. Girls, and Black children, in the United Kingdom are at particular risk for central fatness. Further research is needed to clarify ethnic and other influences on fat distribution, and the health outcomes associated with central fatness.

## Introduction

Ethnic group differences in overweight and obesity, defined by body mass index (BMI) and body fat, have been established (1,2). Waist circumference is an accurate marker of central fatness (3), but research on ethnic differences in this anthropometric measure is more limited, despite growing evidence of its association with metabolic abnormalities (4). Data from the Millennium Cohort Study (MCS) provide a unique opportunity to examine differences in waist circumference in a contemporary, ethnically-diverse, cohort.

## Subjects and methods

The MCS is a prospective study of the social, economic and health-related circumstances of children born in the United Kingdom (UK) at the start of the new century. It employed a stratified clustered sampling design to over-represent children living in disadvantaged areas and from ethnic minority groups. Additional information on the sampling framework has been reported previously (5). Ethics approval was obtained from the South West and London Multi-Centre Research Ethics Committees, UK.

We used waist circumference data (UK Data Archive, University of Essex, UK) from the third sweep of MCS, which took place at age 5 years. This was measured in duplicate to the nearest completed millimeter on bare skin or over light clothing, using a non-elasticated SECA tape (SECA, Hamburg, Germany) positioned horizontally midway between the costal margin and the iliac crest. The correlation between the first and second measurements was 0.98. A third measure was performed if the two measures differed by more than 2 cm. Measures taken over clothing were corrected by subtracting 2 cm (Appendix 1). The average of the two closest measures was used for analysis, expressed as a z-score adjusted for age and sex based on reference data from 8 868 children (aged 3–17). These reference data were collected by the British Standards Institute in 1977 (boys) and 1987 (girls), and represent a nationally representative sample of British children, broadly consistent with the sample used to develop the British 1990 reference data for height and weight (6,7).

In total, 14 403 singleton children were measured at age 5 years (mean [Standard deviation, SD] age: 5.2 (0.25]). Families in which the main survey respondent was not female (n=370) or the child's biological mother (n=41), or there were two cohort children from the same family (n=9) were excluded. Waist circumference data were missing in 314 children or implausible (z-scores > ±5) in 22; ethnicity was not recorded in 72. As some participants had more than one exclusion criterion, the final sample size was 13 590 children (6 658 girls).

Analyses were conducted in STATA/SE 10.0 (Stata Corporation, TX), using survey and non-response weights to account for the sampling design and attrition between contacts. Multivariable regression was used to test the influence of potential confounding factors (mother's highest academic qualification, maternal socio-economic status, or number of children in household) on sex and ethnic differences in waist circumference.

## Results

The mean waist circumference of the cohort was 53.7 cm (SD: 4.3) and the mean z-score 0.5 (SD 1.1), indicating a greater waist circumference than the reference population. The waist circumference z-score for girls was marginally larger than for boys (Tables I and II).

**Table I:** Waist circumference summary statistics according to sex and ethnic group

Variables	N	Mean measure cm (SD*)	Mean z-score (SD)
Sex			
Boys	6 932	53.9 (4.3)	0.50 (1.11)
Girls	6 658	53.4 (4.4)†	0.56 (1.10)†
Child's ethnicity			
White	11 576	53.7 (4.1)	0.55 (1.02)
Indian	328	52.6 (6.6)‡	0.15 (1.67)‡
Pakistani	548	52.5 (6.4)‡	0.12 (1.68)‡
Bangladeshi	207	53.3 (7.7)	0.32 (1.91)‡
Black	412	54.9 (6.2)‡	0.78 (1.44)‡
Mixed/Other	519	53.2 (4.9)	0.36 (1.24)‡

* Standard Deviation.

† Significantly different from boys (p<0.05).

‡ Significantly different from White (p<0.05).

**Table II:** Influence of sex and ethnicity on waist circumference z-scores

	Adjusted regression coefficients (95% Confidence Intervals)		
	
Variables	All children	Boys	Girls
Sex			
Boys	0		
Girls	0.07 (0.02, 0.11)		
Child's ethnicity			
White	0	0	0
Indian	−0.39 (−0.59, −0.19)	−0.45 (−0.81, −0.09)	−0.33 (−0.57, −0.10)
Pakistani	−0.38 (−0.53, −0.24)	−0.52 (−0.72, −0.33)	−0.25 (−0.41, −0.09)
Bangladeshi	−0.20 (−0.42, 0.02)	−0.29 (−0.54, −0.04)	−0.10 (−0.42, 0.22)
Black	0.24 (0.06, 0.41)	0.16 (−0.05, 0.37)	0.33 (0.15, 0.51)
Mixed/Other	−0.20 (−0.33, −0.06)	−0.24 (−0.44, −0.03)	−0.16 (−0.34, 0.02)

* Adjusted for mother's highest academic qualification (collected at 9 months in the MCS), maternal socio-economic status (collected at 9 months), and the number of children in household (collected at age 5).

Black children had larger waist circumference than white children, while those of all other ethnic groups had smaller waists (Table I). Its variance was larger in all ethnic minority groups relative to White children. Following adjustment for confounding factors, ethnic differences remained (Table II). Analyses stratified by sex revealed that Indian, Pakistani and Bangladeshi boys, and those from Mixed/Other ethnic groups, had smaller waists than White boys. Indian and Pakistani girls had smaller waists than White girls, while Black girls had larger waists. Further adjustment for body mass index removed these ethnic differences (data not shown), reflecting ethnic differences in overall adiposity.

## Discussion and conclusions

Children in this cohort had larger waist measurements than the reference population dating from the 1970s and 1980s, suggesting an increase in central fatness and not just general fatness assessed with BMI (8). Nevertheless, the reference population were predominantly Caucasian (6), perhaps explaining some of this difference. However, our findings do suggest that waist circumference has risen more steeply in girls than boys, indicating that they are at greater risk of this central fatness, even at a young age. This study found clear evidence of high levels of central fatness in one particular ethnic group, namely, Black children. Although waist circumference was not greater in South Asian children, body fat percentage measured by dual-energy x-ray absorptiometry has been reported to be highest in this group (2).

While there is increasing evidence of ethnic differences in obesity and other measures of body composition, this remains limited and inconclusive. We have previously reported that Black UK children have the greatest infant weight gain (9) and are among those most likely to be obese or overweight, as determined by BMI during the preschool years (1,2,10). Our findings are comparable with evidence from the United States reporting more central adiposity in Black, than White, adolescent females (11); they do, however, differ to those from Sisson et al. (12) who reported significantly higher waist circumference measurements in White, than Black, boys and no ethnic differences in girls, which may reflect sample differences and factors controlled for in our respective analyses.

This is, to our knowledge, the first population-based study of ethnic differences in waist circumference in the UK, using trained interviewers and standardised protocols. The high correlation (0.98) between repeat measurements supports this approach. In this study we have also incorporated and documented a suitable approach to the size adjustments needed when measures are taken over clothing, which could be used in future epidemiological research (Appendix 1).

Further research is needed to examine ethnic differences in body composition. While BMI is a reasonable proxy for body fatness at a population level, it is less reliable when examining ethnic group differences (13), suggesting that a range of anthropometric measurements, including waist circumference, should be used. Future studies should focus on other genetic and environmental risk factors for central fatness in early childhood, as well as its association with adverse health outcomes in the short and longer term.
