# Supplementary material for: Sex and ethnic differences in the waist circumference of 5-year-old children: Findings from the Millennium Cohort Study
Source: Int J Pediatr Obes. 2011 Jun;6(2Part2):e196–8. doi: 10.3109/17477166.2010.526224 (PMC3465805; doi:10.3109/17477166.2010.526224)
Supplement: Supplementary file 1 [file ijpo0006-e196-SD1.pdf]

Supplementary material for: Griffiths LJ, Dezateux C, Cole TJ. Sex and ethnic differences in the waist circumference of 5-year-old children: Findings from the Millennium Cohort Study, Int J Pediatr obes, 2011; 6: e196–e198.

Appendix 1. Justification for 2 cm clothing adjustment.

Assume that the waist is a circle of radius r, and that the effect of wearing clothing increases the radius to $r + d$. The corresponding circumferences are $2\pi r$ and $2\pi(r + d)$, and the increase in circumference due to the clothing is $2\pi d$. The observed excess waist circumference due to clothing in the MCS was 2.0 cm after adjustment (unpublished data), so that $2 = 2\pi d$ and $d = 2/ 2\pi$ or 0.3 cm. Consequently, the observed clothing effect of 2 cm is explained by the clothing being just 3 mm thick.
